# Effects of *Penicillium chrysogenum* var. *halophenolicum* on kraft lignin: color stabilization and cytotoxicity evaluation

**DOI:** 10.1007/s13205-016-0414-x

**Published:** 2016-04-13

**Authors:** Marlene Remédios, Filomena A. Carvalho, Francisco J. Enguita, Carlos Cardoso, Ivo C. Martins, Nuno C. Santos, Ana Lúcia Leitão

**Affiliations:** 1 Departamento de Ciências e Tecnologia da Biomassa, Faculdade de Ciências e Tecnologia, Universidade NOVA de Lisboa, Quinta da Torre, Campus de Caparica, 2829-516 Caparica, Portugal; 2 Instituto de Medicina Molecular, Faculdade de Medicina, Universidade de Lisboa, Av. Prof. Egas Moniz, 1649-028 Lisbon, Portugal; 3 Instituto Nacional do Mar e Atmosfera, Unidade de Valorização dos Produtos da Pesca da Aquicultura, L-IPIMAR, IP-INRB, Av. Brasília, 1449-006 Lisbon, Portugal

**Keywords:** *Penicillium chrysogenum* var. *halophenolicum*, Color, Lignin removal, Toxicity, Transformation

## Abstract

Wood industries and agricultural crops generate an inexhaustible supply of by-products like lignin, which constitutes an environmental problem. Increasing efforts have been done to find new applications for lignin. One of them is as a food additive, but its chemical nature makes it sensitive to browning which constitutes a major drawback for this type of lignin application. In the present study we are documenting how color stabilization of a commercial kraft lignin was achieved after the treatment with *Penicillium chrysogenum* var. *halophenolicum.* In addition the fungal capacity to remove lignin is studied together with the effect of its treatment on cytotoxicity of lignin. *P. chrysogenum* var. *halophenolicum* was able to transform lignin, ensuring its color stability for more than 24 months. Dynamic light scattering and atomic force microscopy showed that the fungus contributed to homogenize particle size and hydrodynamic properties in lignin suspensions without increase the toxicity over HeLa cells and human primary fibroblasts. These findings suggest new uses for kraft lignin after *P. chrysogenum* var. *halophenolicum* treatment providing an effective approach for improve color stability.

## Introduction

Lignin is the second most abundant natural product of vegetal origin, conferring impermeability and structural support to plants (Baurhoo et al. 2008; Pérez and Moraleda-Muñoz 2011). Its high-molecular weight structure is generally achieved by dehydrogenative polymerization of three primary hydroxycinnamyl alcohols (monolignols): coniferyl alcohol (guaiacyl propanol, G), coumaryl alcohol (*p*-hydroxyphenyl propanol, H), and sinapyl alcohol (syringyl propanol, S), being classified as a three-dimensional amorphous polymer. As a major component of lignocellulosic material, lignin is found in wastes that are produced in large amounts by many industries including forestry, agriculture and food. Global wood consumption is around 3.5 × 10^9^ m^3^/year, being broadly applied for pulp and paper products, production of fuel and building materials (Martinez et al. 2005). This huge production generates a large amount of by-products with negative impacts to the aquatic, and consequently to the terrestrial ecosystem. The adverse effect is due not only to the organic load and toxicity of effluents, but also to the esthetically unacceptable color of water bodies largely due to lignin and its derivatives. The brownish dark coloration of water bodies could be the result of enzymatic activities over aromatic compounds present in lignocellulose-derived materials. Among them, polyphenol oxidases catalyze the oxidation of *ortho*-dihydroxyl phenols in the presence of molecular oxygen to the corresponding *ortho*-quinones, which spontaneously polymerize to produce dark or brown pigments (Walker 1977).

One of current challenges faced by a more environmental-friendly science and industry is to employ natural resources and waste products maximizing economic performance. Indeed, new uses for lignin as fuel, biomaterials, biocides, biostabilisers, crop cultivations and animal feed is now a reality (Lora and Glasser 2002). Moreover, it was suggested that purified lignins may also bring new health benefits to animals (Baurhoo et al. 2008). However, the color stability of lignin products constitutes a major drawback for its general use as additive. In this context, the color preservation of food products during processing and storage is one of the main challenges of food industry; since their organoleptic properties are an important requisite that contributes decisively to food acceptance, based on the initial perception of food condition and degree of processing, among other characteristics (Lopez-Nicolas and Garcia-Carmona 2007).

Due to its aromatic nature and structural complexity, lignin is resistant to the attack of the majority of microorganisms (Baurhoo et al. 2008; Pérez and Moraleda-Muñoz 2011). Indeed, in nature, white-rot fungi are the only efficient lignin degraders, since they have the ability to completely mineralize this polymer, and consequently they are quite important in the global turnover of carbon from woody plants (Wong 2009). The best studied are *Phanerochaete chrysosporium*, *Pleorotus ostreatus*, *Bjerkandera adusta*, *Pycnosporus cinnabarinus*, *Ceriporiopsis subvermispora*, *Phlebia* sp., or *Trametes versicolor* (Pérez and Moraleda-Muñoz 2011). Meanwhile, there are other fungi that have the ability to partially degrade lignin. For instance, a *Penicillium chrysogenum* strain was able of using kraft, organosolv, and synthetic dehydrogenative polymerised lignins with low degradation rates (Rodriguez et al. 1994). Despite of that, only a few reports have discussed lignin degradation by *Penicillium* strains (Polman et al. 1994; Rodriguez et al. 1994; Hao et al. 2006; Yadav and Yadav 2006; Dwivedi et al. 2011); however, as far as we know kraft lignin removal and color stabilization by imperfect fungi has not yet been described. We have previously characterized a halotolerant *P. chrysogenum* strain, *P.* *chrysogenum* var. *halophenolicum*, isolated from a salt mine (Leitão et al. 2012), and able to metabolize phenolic compounds under osmotic stress (Leitão et al. 2007; Guedes et al. 2011).

In the present work, we aimed to study the effect of *P. chrysogenum* var. *halophenolicum* on kraft lignin transformation and color stability, investigating if this strain has the potential to become a browning control agent, to impart lignin with stable color that may promote its use as an additive for biotechnological applications.

## Materials and methods

### Strain and culture conditions

*P. chrysogenum* var. *halophenolicum* was used throughout this study; this strain was isolated from a salt mine in Algarve, Portugal, and previously characterized (Leitão et al. 2012).

*P. chrysogenum* var. *halophenolicum* was maintained at 4 °C on nutrient agar plates (Difco, BD diagnostic systems, Hunt Valley, MB, USA) with 2 % (w/v) NaCl. Pre-cultures of cells were routinely aerobically cultivated (160 rpm in a Certomat^®^ BS-T Incubator, Sartorius stedim biotech, Goettingen, Germany) at 25 ± 1 °C in 100 mL of complex medium (MC: glucose, 30.0 g/L; NaNO_3_, 3.0 g/L; MgSO_4_·7H_2_O, 0.5 g/L; NH_4_Fe(SO_4_)_2_·12H_2_O, 10.0 mg/L; K_2_HPO_4_, 1.0 g/L; yeast extract, 5.0 g/L; NaCl, 20.0 g/L; pH 5.6).

To investigate the ability to transform kraft lignin, the strain was cultivated in 500-mL flasks containing 100 mL of MC during 68 h. Cells were collected by centrifugation and washed in 0.85 % (w/v) of NaCl. A 15 % of the pre-inoculum was inoculated in modified Janshekar medium (Janshekar et al. 1982) containing NaNO_3_ (1.00 g) in substitution of NO_3_NH_4_ (0.496 g) and without vitamin solution and nitrilotiacetate as a component of trace element solution and amended with 1700 mg/L kraft lignin (alkali, low sulfonate content was obtained from Sigma-Aldrich, St. Louis, MO, USA). *P. chrysogenum* var. *halophenolicum* was aerobically incubated (160 rpm in the Certomat^®^ BS-T Incubator) in the dark during 96 h, at 25 °C. Three replicates were used. Abiotic assays were performed in parallel with uninoculated flasks (duplicates) as negative controls.

After regular times of culture, cells were harvested and 12 mL of supernatant were kept frozen at −40 °C until lignin determination assays, while approximately 12 mL were maintained at room temperature for lignin color stability experiments.

Microbial dry biomass was estimated gravimetrically by the method described by Gunther et al. (1995).

### Lignin quantification and color stability evaluation

The commercial kraft lignin is an ideal substrate for assessing color stability and biological transformation of lignin. Firstly, the use of commercial alkali lignin avoids the concerns about chemical composition, the isolation method, or purity, since it is a commercial product. Secondly, commercial alkali lignin was employed in other studies to assess chemical effect on the structure of lignins (Kadam and Drew 1986; Suparno et al. 2005; Yuan et al. 2010; DeAngelis et al. 2011; George et al. 2011; Huang et al. 2013).

Kraft lignin color stability experiments were conducted in a lab room fitted with a chamber temperature set at 22 °C without any special storage condition. After a time of 7, 50 days and 7 months, samples were filtered before color measurement, as described in the section of color measurement. All assays were performed in *n* = 3 repetitions. The mean values and standard deviations were evaluated using analysis of variance (ANOVA).

Lignin concentrations were quantified by spectrophotometric absorption at 205 nm in a Spekol^®^ 1500 UV VIS Spektralphotometer (Analytic Jena AG, Germany).

### Atomic force microscopy

A NanoWizard II atomic force microscope (JPK Instruments, Berlin, Germany), mounted on the top of an Axiovert 200 inverted microscope (Carl Zeiss, Jena, Germany) was used for imaging the samples. The AFM head is equipped with a 15-μm z-range linearized piezoelectric scanner and an infrared laser. Samples were diluted to 1:200 in Milli-Q water and deposited on freshly cleaved muscovite mica for 20 min. After subsequent washes, the sample was allowed to air dry at room conditions. Imaging of the samples components were performed in air tapping mode (for the 1st day fungal treated samples) and in contact mode (for the 4th day fungal treated samples). Oxidized sharpened silicon tips (ACL tips from Applied Nanostructures, CA) with a tip radius of 6 nm, resonant frequency of about 190 kHz and spring constant of 45 N/m were used for the measurements. Imaging parameters were adjusted to minimize the force applied on the scanning of the topography of the samples. Scanning speed was optimized to 0.4 Hz (on the first day of treatment sample) and 0.8 Hz (on the fourth days of treatment sample), and acquisition points were 512 × 512 and 360 × 360, respectively. Imaging data were analyzed with the JPK image processing v.3 (JPK Instruments). The width and height of the samples were calculated from the cross section plots. All complex dimensions measurements were performed using the Gwyddion software (Czech Metrology Institute, Brno, Czech Republic), version 2.19.

### Dynamic light scattering (DLS) measurements

Dynamic light scattering experiments were carried out at 25 °C on a Malvern Zetasizer Nano ZS (Malvern, UK), with a backscattering detection at 173°, equipped with a He–Ne laser (*λ* = 632.8 nm), using glass cuvettes with round aperture. Kraft lignin samples were diluted 1/10 in MiliQ water The samples were left equilibrating for 15 min at 25 °C before each measurements set (10 measurements; each one being the average of 10 runs, with 10 s per run). Normalized intensity autocorrelation functions were analyzed using the CONTIN method (Provencher 1982a, b), yielding a distribution of diffusion coefficients (*D*). The measured *D* (m^2^/s) was used for the calculation of the hydrodynamic diameter, *D*_H_ (nm), through the Stokes–Einstein relationship (Berne and Pecora 1990):

$$ D_{\text{H}} = \frac{\kappa T}{3\pi \eta D} $$

where *κ* is the Boltzmann constant (J/K), *T* the absolute temperature (K), and *η* the medium viscosity (Pa s). The data were statistically analyzed within each set of 10 measurements by observing the average and standard deviation, and discarding outliers. The average without outliers became close to the median in all the size points.

### Color measurement

Samples color was determined using a Macbeth eye 3000 colorimeter. The color in the CIELAB system is characterized by three parameters, L*, a* and b*. The lightness value (L*), taking values from 0 % (black) to 100 % (white), a* from green (−*a*) to red (+*a*) and b* from blue (−*b*) to yellow (+*b*). The L*, a* and b* color coordinates of each group of samples were measure after stability experiment. These values were then used to calculate the color change ΔE* as a function of the stability experiment duration according to the following equations:

$$ \Delta L^{*} = L^{*}_{f} - L^{*}_{i} $$

$$ \Delta a^{*} = a^{*}_{f} - a^{*}_{i} $$

$$ \Delta b^{*} = b^{*}_{f} - b^{*}_{i} $$

$$ \Delta E^{*}  = (\Delta L^{{*2}}  + \Delta a^{{*2}}  + \Delta b^{{*2}} )^{{1/2}}  $$

where ΔL*, Δa* and Δb* are the changes between the initial (*i*) and the final (*f*) values and ΔE* corresponds to a color change. The color evolution assays were performed using the measurements at time 7 days as standards, which correspond to the first measurement (*i*) that was made after the fungal treatment, mycelia removal and storage at 22 °C.

### Cytotoxicity evaluation

Quantification of the toxic effects of kraft lignin and fungal treated samples at the cellular level was performed by the use of Alamar Blue^®^ test to quantify HeLa and human primary skin fibroblasts (Coriell Instutute for Medical Research, ref. GM05565) viability (Invitrogen) using a modified version of an already described protocol (Al-Nasiry et al. 2007). Briefly, HeLa and fibroblast cells were plated in 96 well plates at 10^5^ cells per well in RPMI medium containing 10 % FBS, and incubated at 37 °C and 5 % CO_2_ during at least 8 h to allow cell attachment to the plate surface. After this incubation time, RPMI was substituted by Opti-MEM medium to avoid serum interference with the assay. Cells were then exposed to different dilutions of the *P. chrysogenum* var. *halophenolicum* culture supernatants, previously buffered with 10 × PBS and sterilized by filtration though 0.22 µm membranes. After 48 h, HeLa and fibroblast cells were washed twice with PBS and incubated during 1 h at 37 °C with Alamar Blue^®^ following the recommendations from the manufacturer. Cell viability was quantified by determination of the fluorescence of Alamar Blue^®^ at 590 nm after excitation at 530 nm in a fluorescence plate reader (TECAM Infinite M200). EC_50_ were determined by non-linear regression using an equation for a sigmoid curve (GraphPad Prism 5.02).

## Results and discussion

### Lignin removal

**Fig. 1:**
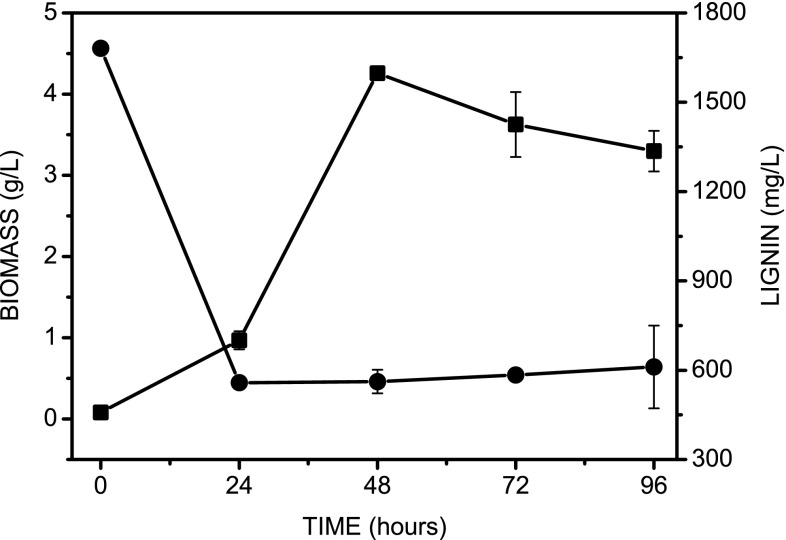
Kraft lignin removal by *P. chrysogenum* var. *halophenolicum* during 96 h under aerobic conditions. *Squares* cell growth; *circles*, kraft lignin concentration. Data shown represents average of triplicates ± standard deviations

To determine the ability of *P. chrysogenum* var. *halophenolicum* to remove kraft lignin, the microorganism was cultured in the presence of 1700 mg/L of commercial kraft lignin. Since no abiotic loss of lignin was detected in control samples, the decrease of lignin concentration in the presence of fungus must be due to its biological action. The growth of *P. chrysogenum* var. *halophenolicum* and lignin removal is depicted in Fig. 1. At the initial concentration of 1700 mg/L of kraft lignin no lag phase was observed. A lignin removal of 68.0 % was achieved after 96 h of fungal treatment, showing a clear correlation with fungal growth. Indeed, data indicate that there were two peaks in biomass, corresponding to exponential growth phase. The first one should be due to the presence of glucose in the culture media composition besides lignin, since the fungal inoculum was prepared in a complex medium with glucose, and its enzymatic system was already active and ready to use this substrate. This fact was corroborated by the absence of lag phase on fungal growth. After 24 h of culture, a significant increase on fungal biomass and a concomitant decrease in the lignin concentration were observed, indicating that *P. chrysogenum* var. *halophenolicum* growth was due to lignin transformation. In general, most of ligninolytic fungi reported require a high level of consumption of easily metabolized cosubstrate to be effective in the ligninolysis. The *P. chrysogenum* var. *halophenolicum* was able to remove more than 1000 mg/L of kraft lignin in the first 24 h of batch culture, which is quite interesting; whereas the heteropolymer concentration did not significantly decreased during the last three days of fungal treatment, probably due to the C–C bonds of alkaline lignin that are highly resistant to hydrolytic breaking (Yuan et al. 2010). Kraft lignin is enriched with guaiacyl groups, where the C5 position in the aromatic ring represents the most abundant C–C linkage in lignin molecules (El Mansouri et al. 2011). Fungi are the only microorganisms extensively studied for the degradation of lignin. A strain of *Penicillium chrysogenum* is able to degrade some synthetic and natural lignins, and mineralize 7.9 % of [^14^C_β_] DHP in 29 days (Rodriguez et al. 1996).

Recently several studies were published using commercial kraft lignin. Among them, Huang et al. reported that 500 and 700 mg/L of commercial kraft lignin is degraded by *Bacillus pumilus* and *Bacillus atrophacus*, respectively, after 18 days of incubation (Huang et al. 2013). A comparative study of natural and commercial kraft lignin degradation by *Citrobacter* sp. was described, showing its higher capacity to use kraft lignin than commercial kraft lignin (252 and 186 mg/L, respectively) (Chandra and Bharagara 2013). Our results indicate that *P. chrysogenum* var. *halophenolicum* efficiently removed kraft lignin.

### Effect of fungal treatment on the evolution of lignin color

To evaluate the effect of *P. chrysogenum* var. *halophenolicum* on lignin color, scalar parameters (L*, a*, and b*) were determined, after 7 days of storage, in samples with different times of fungal treatment. The initial L* value for untreated sample was 56.45. Fungal treatment did not induce any significant change in L* values (Table 1). After 96 h of fungal treatment the L* value was 56.88, indicating that the components absorbing visible light remain constant.

**Table 1:** Evolution of L* and a* and b* coordinates of kraft lignin samples after the absence and presence of *P. chrysogenum* var. *halophenolicum.* After treatment, fungal micelia were removed and the samples were stored at 22 °C during 7 days before color estimation (*n* = 3)

Fungal treatment (h)	L*	a*	b*
Control	56.45 ± 0.04 a	−0.81 ± 0.03 a	1.87 ± 0.03 a
24	56.53 ± 0.22 a	−0.74 ± 0.02 a	2.10 ± 0.15 ab
48	56.82 ± 0.02 b	−0.91 ± 0.06 ab	2.38 ± 0.01 c
72	56.64 ± 0.10 a	−0.98 ± 0.02 b	2.18 ± 0.02 b
96	56.88 ± 0.05 b	−0.87 ± 0.04 a	2.47 ± 0.03 c

Different letters (vertically) indicate significant differences (*p* < 0.05)

In the samples treated with the fungus during 24 h the a* values slightly increased, possibly due to the presence of degradation and/or oxidation products, followed by a decrease at later culture times. Nevertheless, the different times of culture led to lower values of a*, with the initial green color still remaining after 96 h of treatment (Table 1). These results are compatible with chemical changes of lignin.

The b* values of samples increase after fungal treatment. The increase in yellowness could be caused by the low-molecular-weight phenolic compounds obtained by the action of fungal enzymes.

### AFM studies

**Fig. 2:**
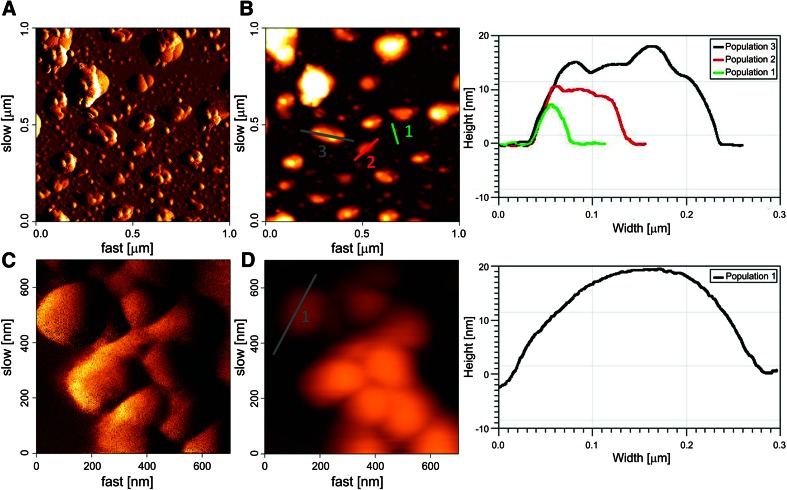
AFM scanning images of kraft lignin with 1 and 4 days of treatment with *P. chrysogenum* var. *halophenolicum*. Air tapping mode AFM error (**a**) and height (**b**) images of kraft lignin with 1 day of fungal treatment (*horizontal scale* 1 μm × 1 μm; height scale up to 27.6 nm). Air contact mode AFM error (**c**) and height (**d**) images of kraft lignin with 4 days of fungal treatment (horizontal scale: 700 nm × 700 nm; height scale up to 66.5 nm). Cross section analysis of the AFM height images could also be performed. Three examples of cross sections of the different visualized populations (**b** *right image*) and one example of a homogeneous population (**d**, *right image*) of the kraft lignin profiles are shown. With this type of analysis, lignin size, height, shape and roughness can be determined

In nature, lignin is an amorphous polymeric material often associated to cellulose fibers as main constituents of plant cell walls. Since the exact structure of lignin itself is not known (Chakar and Ragauskas 2004; George et al. 2011), atomic force microscopy has been applied to characterize the topography and roughness of lignocellulosic materials in their natural forms (Chundawat et al. 2011). The AFM scanning images of lignin samples with one (a, b) and 4 days (c, d) of treatment with *P. chrysogenum* var. *halophenolicum* are shown on Fig. 2.

From the figure, it can be evidently noticed that fungal treatment on the lignin samples leads to morphological changes within the polymer. From air tapping mode AFM error (a) and height (b) images of the sample treated through only 24 h, it could be seen that it is very heterogeneous in size. Opposing this, the lignin sample with 96 h of treatment is much more homogenous and only one size population could be visualized. To quantitatively analyze AFM height images of these populations, we measured the diameter and the height of their surface profiles (Fig. 2b, d, right images). Cross section analysis of each feature on the images was done, yielding distinct profiles. Examples of these cross sections are also shown (Fig. 2b, d, left images). The analysis indicates that the sample with 96 h of treatment had a unique population, with an average diameter of 321.2 ± 53.7 nm and height of 28.8 ± 8.6 nm (*n* = 11). We could distinguish three different populations on the lignin sample with only 24 h of fungal treatment, with different dimensions: 42.5 ± 6.0 nm (diameter) and 5.1 ± 1.0 nm (height) on population 1 (*n* = 9); 124.2 ± 17.3 nm (diameter) and 16.8 ± 2.7 nm (height) on population 2 (*n* = 16); and, 221.9 ± 36.9 nm (diameter) and 34.8 ± 10.9 nm (height) on population 3 (*n* = 23). There were no significant differences in the visualized roughness of both analyzed samples (Fig. 2b, d, right images).

From the calculated dimensions of the three populations of the 24 h treatment sample, we could hypothesize that populations 2 and 3 were formed by oligomers of the lignin structures of population 1. After 96 h of treatment, the substantial increase in size of the lignin particles achieved on the entire population reveals the existence of polymerization or major aggregation phenomena.

### DLS analysis

DLS experiments of untreated lignin and fungal treated samples are shown in Table 2. Untreated lignin showed a great variation in particle sizes and poorly resolved peaks, with three major ones observed, one corresponding, roughly, to 6–20 nm sized species, the other to 20–1000 nm sized species and a final one corresponding to species well over 3 µm. Taking only the first two (which are better resolved), the maxima of the peaks correspond to average hydrodynamic diameters (*D*_H_) of 16.6 nm (scattering intensity 34.4 %) and 629.7 nm (scattering intensity 65.6 %). Upon 24 and 48 h of treatment with the fungus, the sample sizes become more uniform and overall smaller. After 24 h of treatment three major peaks were observed, but better resolved than in the control, since the first one corresponds mostly to, approximately, 10–20 nm sized species, the second one to 150–400 nm sized species and the third one to species with more than 2 µm. Taking again only the first two (which are much better resolved), the maxima of the peaks corresponded to average hydrodynamic diameters (*D*_*H*_) of 15.5 nm (scattering intensity 44.9 %) and 301.3 nm (scattering intensity 42.3 %). The treatment with *P. chrysogenum* var. *halophenolicum* appeared to eliminate the larger particles (centered around 629.7 nm) resulting in much smaller structures, with about half of the hydrodynamic diameter of the control samples (hydrodynamic diameter centered around 301.3 nm). These results support the hypothesis that the fungus had the ability to use differently sized lignin molecules, as described recently by Wang et al. in the lignosulfonate biodegradation process by *Sphingobacterium* sp. HY-H (Wang et al. 2013). The third peak did not have a good resolution, however, it is clear that the overall scattering particles size decreased, and that also in parallel, the amount of particles with sizes above 1 µm is diminished, resulting on an overall extensive reduction and uniformization in particles size.

**Table 2:** Average particles size of fungal treatment samples (hydrodynamic diameter) estimated by dynamic light scattering (DLS)

Incubation time (h)	Samples	Peak 1	Peak 2	Peak 3
24	Fungal treatment	15.5 nm (44.9 %)	301.3 nm (42.3 %)	3590.0 nm (12.8 %)
	Control	16.6 nm (34.4 %)	629.7 nm (65.6 %)	No major peak
48	Fungal treatment	15.2 nm (5.0 %)	191.5 nm (80.6 %)	3551.0 nm (12.8 %)
	Control	17.0 nm (29.5 %)	718.4 nm (64.0 %)	4202.0 nm (6.5 %)
96	Fungal treatment	No peak	245.7 nm (92.4 %)	4397.0 nm (7.6 %)
	Control	13.0 nm (35.3 %)	250.5 nm (43.0 %)	2053.0 nm (21.7 %)

Size represents the peak average based on size distribution by volume; the percentages represent the peak area, dark shadowed area highlights homogeneously sized samples (e.g., samples with over 80 % of the particles included within a single size distribution peak)

The same profile was observed after 96 h of treatment, but in such an extent that the sample can be said to be essentially homogeneous, with one peak at 245.7 nm (scattering intensity 92.4 %), with observed reasonable agreement with the AFM data (321.2 ± 53.7 nm). A small unresolved peak of particles with more than 1 µm was also visible, but with a limited fraction of the intensity. Meanwhile, in the untreated samples three peaks clearly remains after 96 h. These results were consistent with the hypothesis that the presence of *P. chrysogenum* var. *halophenolicum* could promote the polymerization of lignin into well-defined and homogenously sized samples. Overall, and in spite of the small differences between observed AFM and DLS particles size due to the different precision level of the two techniques (DLS size estimation may be distorted by larger particles, while the flattening of the sample to a discoidal shape on the AFM substrate and/or convolution with the AFM tip dimensions also affects size determinations), the results obtained using AFM topographic images were in accordance with those achieved via DLS, showing that not only the low-molecular-weight organic compounds, but also the polycyclic aromatic compounds can be degraded by *P. chrysogenum* var. *halophenolicum* with the concomitant uniformization of the lignin samples into a single sized species after 96 h of treatment with the fungus.

### Lignin color stability

As described above, DLS and AFM show that, after 96 h of fungal treatment, the lignin samples become highly homogenized in terms of size, estimated to be below 390 nm, the approximate lower limit for light detection by the human eye (Starr 2005). This fact prevents, among other phenomena that may interfere with color stability, the occurrence of light scattering phenomena. This can occur in relatively dense solutions, especially, if they form differently sized particles that might eventually modify the color perception. However, in this particular case, light scattering by these uniformly sized lignin particles (<390 nm) would not be a problem since it would be already in the ultraviolet light spectrum, invisible to the human eye, which helps strengthen the potential of lignin treated samples as color stabilizer. To further evaluate this potential, the lightness values for samples treated with the *P. chrysogenum* var. *halophenolicum* after 50 days and 7 months under stability assay conditions, were comparable or slightly higher than the initial L* values (Table 3). Moreover, color stability increased after 50 days of storage, since the overall color change (Δ*E* *) values of the samples treated with the fungus were smaller than those of the control samples (Table 3).

**Table 3:** Evolution of color difference (ΔE*) after 50 days, 7 and 26 months of storage

Fungal treatment (h)	ΔE*		
	50 days/7 days	7 months/7 days	26 months/7 days
Control	0.59 ± 0.52	1.20 ± 0.62	n.d.
24	0.55 ± 0.19	0.90 ± 0.67	1.06 ± 0.19
48	0.26 ± 0.01	0.60 ± 0.04	n.d.
96	0.17 ± 0.04	0.61 ± 0.05	0.68 ± 0.05

*n.d*. not determined

For treated fungal samples, the color change for 24 h was 0.55. The color change value was 0.17 for 96 h in the treated fungal samples. These results showed a trace visual change in samples treated more than 48 h with *P. chrysogenum* var. *halophenolicum,* according to the scale proposed by Dirckx et al. (Dirckx et al. 1992). Furthermore, the overall color change decreased as fungal contact time increased, suggesting heightened color stability. At the end of 7 months storage, a slight increase of Δ*E* * in the treated samples was observed, when compared to the values obtained at 50 days of storage. Nevertheless, in all samples treated with fungus after 7 months of storage, the overall color change was lower than that of the control with 50 days. Furthermore, in the culture treated during 96 h no significant color change was observed between 7 and 26 months of storage, which is in excellent agreement with the DLS and AFM size estimation showing stable uniform molecules (after fungal treatment). Overall, this strongly supports the hypothesis that the treatment with *P. chrysogenum* var. *halophenolicum* stabilizes the lignin molecules properties, especially in what regards to color and to its possible use as a color modifies/stabilizer.

### Cytotoxicity of kraft lignin before and after fungal treatment

**Fig. 3:**
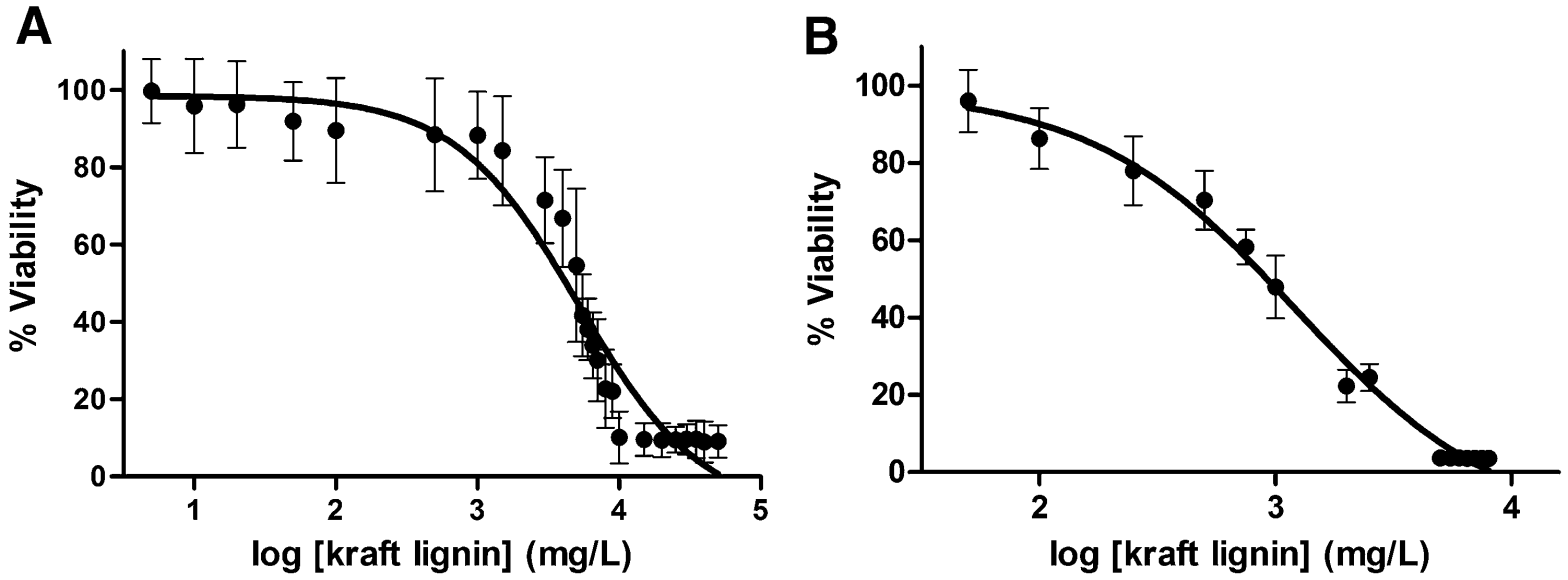
Kraft lignin dose response curves using the Alamar Blue assay. Curves are examples obtained by average of five experimental replicates and illustrate the response of the human primary skin fibroblasts (**a**) and HeLa cells (**b**)

The evaluation of the potential toxic effects of lignin metabolites was conducted by the determination of the relative proportion of viable cells, by the Alamar Blue^®^ assay. This test is based on the continuous conversion by viable cells of resazurin, a non-fluorescent cell permeable pigment, to resorufin, a fluorescent compound that can be determined by fluorimetric assay, yielding a quantitative measurement of cell survival. We used HeLa cells and human primary skin fibroblasts viability as an indicator of the potential toxic effects of lignin and its metabolites. The EC_50_ values of kraft lignin showed that it had cytotoxic effects, but only at very high concentrations (Fig. 3). In incubations with fibroblasts, the EC_50_ for cytotoxicity was higher than HeLa cells (EC_50_ of 5177 ± 1726 and 1223 ± 328 mg/L, respectively in fibroblasts and HeLa cells). At the concentrations of 750, 1000, 2000 mg/L, we observed a significant dose-dependent increment in the HeLa cells viability. A good correlation between the cytotoxic effects on HeLa cells and on fibroblasts cells after 48 h was observed (*r* = 0.9775 and *r* = 0.9509, respectively). As it is previously reported the differences between a cancer cell line and primary fibroblasts can be attributed to differences in cell sensitivity to the compound that is assayed and would be mainly related with the cell division rate (Ugartondo et al. 2008).

**Fig. 4:**
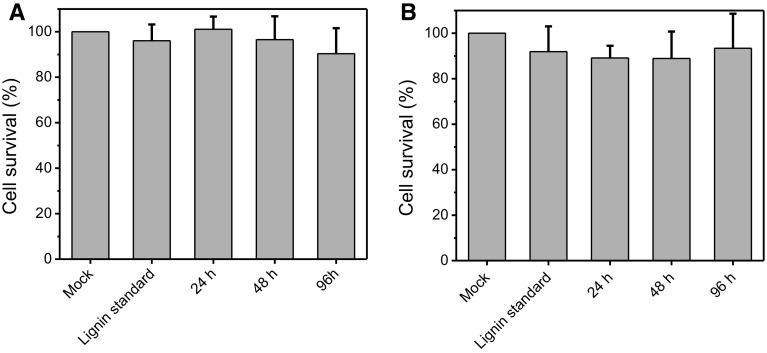
HeLa and fibroblast cells viability quantified by Alamar Blue assay after incubation with fungal treated and untreated kraft lignin preparations. **a** HeLa cells, **b** human primary skin fibroblasts. Mock, untreated cells; Kraft lignin standard, cells incubated with soluble lignin at a concentration of 50 mg/L; 24, 48 and 96 h represent the time of fungal treatment. Cell survival was calculated as the ratio of fluorescence between sample or standard and mock

To assess if lignin metabolites generated from *P. chrysogenum* var. *halophenolicum* biological activity are nontoxic for HeLa and fibroblast cells, new experiments were done. The original samples were diluted in cell culture medium before to cytotoxicity testing to obtain a concentration range similar to those where standard lignin concentration begins to be toxic to the tested cells. Results shown in Fig. 4 clearly indicate that both HeLa and fibroblast cells viability were not significantly modified by the exposure to *P. chrysogenum* var. *halophenolicum,* in comparison with the standard lignin at the same concentrations, indicating that the lignin metabolites generated by *P. chrysogenum* var. *halophenolicum* were not cytotoxic.

These results were quite important since it is reported that many dyes are believed to be toxic, some of them as a result of microbial metabolism (Couto 2009). Additionally, *Penicillium* species are known to produce a high abundance of secondary metabolites including mycotoxins such as ocratoxin A, citrinin, verrucosidin, patulin, among others, which have different degrees of toxicity on target organs or are carcinogenic (Chávez et al. 2011). Therefore, it is important to assess the cytotoxic effects of lignin samples after treated with *P. chrysogenum* var. *halophenolicum* to discard any possible cytotoxic properties.

Despite the potential for biological hazard is low for fungi converted feed as so far, assess cell viability is of major importance to ensure consumer health and safety. The present data indicated that *P. chrysogenum* var. *halophenolicum*, in this test conditions, did not increase lignin toxicity, showing that kraft lignin can be used over an effective concentration range that is safe for normal and cancer cell lines studied.

The environmental protection agencies are becoming more restrictive regarding water discharge from industrial effluents. Several industries, besides the classical primary and secondary treatments, use physical and chemical tertiary treatments to remove the color cause by lignin and lignin derivatives, which are expensive and not always lead to high performance. Biological technology is a feasible and promising alternative. Fungi have attracted a great deal of interest as potential biomass and recalcitrant compounds degraders such as lignin due to their ability to produce a broad diversity of extracellular ligninolytic enzymes some of them with lack specificity for a particular substrate. If white-rot fungi completely mineralize lignin, other fungi and bacteria lead to the production of brown pigments which limits the practical use of such agro residues. Meanwhile, although lignin was generally considered to be nutritionally inert, new scientific data on health-protective mechanisms of cereals endorse the opposite idea (Fardet 2010). Meister advanced a new possible use for modified lignin to pet and human food as roughage, a fiber source, or a cancer protection agent (Meister 2002). Recently, Tortora et al. described the use of kraft lignin as the raw material for microcapsulation processes assembled by ultrasound into lignin microcapsules to storage and release Coumarin-6 (Tortora et al. 2014). Several researchers pointed out that a lignin with a more constant structure and size will enhance its potential utilization in high-value products (Norgren and Edlund 2014; Qu et al. 2015).

In summary, this work showed that *P. chrysogenum* var. *halophenolicum* was able to transform kraft lignin, displaying a higher capacity to grow on lignin substrates. AFM and DLS assays indicated that kraft lignin biotransformation proceeds either via low-molecular phenolic compounds or oligomers. *P. chrysogenum* var. *halophenolicum* treatment resulted in a stabilization of commercial soluble kraft lignin color. It is also remarkable that lignin color stability was recorded for more than 24 months at 22 °C under light conditions of storage. These results were compatible with the hypothesis that *P. chrysogenum* var. *halophenolicum* could be a potential tool for stabilizing lignin against color change by transforming the heterogeneous lignin composition in a more homogenous structure. The increased lignin stability was achieved without increasing toxicity over HeLa and fibroblast cells. These findings could promote the application of *P. chrysogenum* var. *halophenolicum* for the development of lignin-fortified formula that would improve food fiber content.
